# Similarity of Bisphenol A Pharmacokinetics in Rhesus Monkeys and Mice: Relevance for Human Exposure

**DOI:** 10.1289/ehp.1002514

**Published:** 2010-09-20

**Authors:** Julia A. Taylor, Frederick S. vom Saal, Wade V. Welshons, Bertram Drury, George Rottinghaus, Patricia A. Hunt, Pierre-Louis Toutain, Céline M. Laffont, Catherine A. VandeVoort

**Affiliations:** 1 Division of Biological Sciences; 2 Department of Biomedical Sciences and; 3 Veterinary Medical Diagnostic Laboratory, University of Missouri–Columbia, Columbia, Missouri, USA; 4 School of Molecular Biosciences, Washington State University, Pullman, Washington, USA; 5 INRA, TOXALIM (Research Centre in Food Toxicology), Toulouse, France; 6 Ecole Nationale Veterinaire de Toulouse, Université de Toulouse, Toulouse, France; 7 Department of Obstetrics and Gynecology, California National Primate Research Center, University of California–Davis, Davis, California, USA

**Keywords:** biomonitoring, bisphenol A, endocrine disruption, pharmacokinetics, xenobiotic metabolism

## Abstract

**Objective:**

Daily adult human exposure to bisphenol A (BPA) has been estimated at < 1 μg/kg, with virtually complete first-pass conjugation in the liver in primates but not in mice. We measured unconjugated and conjugated BPA levels in serum from adult female rhesus monkeys and adult female mice after oral administration of BPA and compared findings in mice and monkeys with prior published data in women.

**Methods:**

Eleven adult female rhesus macaques were fed 400 μg/kg deuterated BPA (dBPA) daily for 7 days. Levels of serum dBPA were analyzed by isotope-dilution liquid chromatography–mass spectrometry (0.2 ng/mL limit of quantitation) over 24 hr on day 1 and on day 7. The same dose of BPA was fed to adult female CD-1 mice; other female mice were administered ^3^H-BPA at doses ranging from 2 to 100,000 μg/kg.

**Results:**

In monkeys, the maximum unconjugated serum dBPA concentration of 4 ng/mL was reached 1 hr after feeding and declined to low levels by 24 hr, with no significant bioaccumulation after seven daily doses. Mice and monkeys cleared unconjugated serum BPA at virtually identical rates. We observed a linear (proportional) relationship between administered dose and serum BPA in mice.

**Conclusions:**

BPA pharmacokinetics in women, female monkeys, and mice is very similar. By comparison with approximately 2 ng/mL unconjugated serum BPA reported in multiple human studies, the average 24-hr unconjugated serum BPA concentration of 0.5 ng/mL in both monkeys and mice after a 400 μg/kg oral dose suggests that total daily human exposure is via multiple routes and is much higher than previously assumed.

In 1936, bisphenol A (BPA) was reported to have the activity of an estrogenic drug (Dodds and Lawson 1936). Today, BPA is used in a large number of consumer products and is one of the highest volume chemicals produced, on the order of 8 billion pounds per year (Bailin et al. 2008). A large body of evidence now indicates that BPA is an endocrine-disrupting chemical that can induce a variety of adverse effects in mammals and other vertebrates and invertebrates (Oehlmann et al. 2008; Richter et al. 2007), but its safety continues to be disputed (Goodman et al. 2009). Much remains to be determined about the mechanisms of action of BPA, which varies depending upon the dose, tissue, and life stage of exposure (vom Saal et al. 2007), but understanding the current levels of human exposure and the various routes of exposure to BPA, how BPA is metabolized, and whether animal models are relevant for modeling human exposure is critical to better understand the risk posed to humans. The urgent need for this information is underscored by the Centers for Disease Control and Prevention’s conclusion that > 90% of people in the United States are chronically exposed to BPA (Calafat et al. 2008) and the suggestion that this likely also applies to people living in other countries around the world (Vandenberg et al. 2010a).

Surprisingly, no available data directly bear on the question regarding sources and amounts of human exposure to BPA, and estimates of current daily BPA exposure levels vary widely. The U.S. Food and Drug Administration (FDA) estimated that the daily BPA exposure level for adults in 2007 was about 0.16 μg/kg/day (FDA 2008). However, after reviewing BPA levels reported in all available studies of human tissues, scientists at a 2007 conference sponsored by the National Institute of Environmental Health Sciences predicted that exposure levels of > 35 mg/day (~ 500 μg/kg/day) would be required to account for the reported levels of BPA in adults (Vandenberg et al. 2007; vom Saal et al. 2007). This information was recently updated (Vandenberg et al. 2010a), and the models used for calculating human exposure, as well as the assumption that virtually all BPA exposure is entirely from food and beverage containers, were sharply criticized (Gies et al. 2009; Vandenberg et al. 2010b).

Our understanding of current levels of human BPA exposure is complicated by our limited knowledge of the ways by which we are exposed. Because BPA leaches into food from plastic packaging and resin linings of food and beverage containers, it has been widely assumed that the consumption of contaminated food and beverages represents the major route of human exposure. However, new sources of exposure continue to be uncovered, such as thermal (carbonless) receipts used for many daily transactions that contain a coating of high levels of free BPA, raising the possibility that dermal transport may also be a significant source of exposure (Biedermann et al. 2010; Environmental Working Group 2010). There is significant leaching of BPA from children’s books (Sajiki et al. 2010), and BPA is also present in cigarette filters, raising the concern that inhalation of cigarette smoke may be another previously unrecognized source of exposure for individuals who smoke (Jackson and Darnell 1985).

In the absence of human pharmacokinetic data for unconjugated (bioactive) BPA, findings from studies in rodents and monkeys have been used to extrapolate to humans. The low BPA doses used in rodent studies lead to serum levels of unconjugated BPA significantly below levels found in biomonitoring studies of men and pregnant and nonpregnant women (Vandenberg et al. 2007), yet these low internal levels of BPA have been reported to result in numerous developmental abnormalities (Richter et al. 2007). However, it has been argued that major metabolic differences between humans and rodents preclude extrapolation of these data to humans (Dekant and Völkel 2008). Specifically, although glucuronidation of BPA by uridine 5′-diphospho (UDP)-glucuronosyltransferases (UGTs) is a primary mode of phase II metabolism in both rodents and primates, in adult primates BPA is cleared from the blood by the kidney into the urine (Figure 1), whereas in rodents the primary excretory pathway for BPA is via the bile into the feces (Inoue et al. 2005; Sakamoto et al. 2002). There may be other metabolic differences between species; in the CD-1 mouse, glucuronidation includes the glucuronidation of both BPA and hydroxylated BPA (Zalko et al. 2003), but data on this are lacking for primates. The species differences in route of clearance have been interpreted as indicating that the value for clearance of unconjugated (bioactive) BPA must also be very different between rodents and primates.

To date, only a single study has attempted to examine BPA clearance rate from blood after a single oral administration in adult humans (Völkel et al. 2002). However, because the assay used in that study was not sufficiently sensitive to measure unconjugated serum BPA, only the concentration of conjugated BPA in human serum was reported. The study by Völkel et al. (2002) has been repeatedly cited as evidence for rapid clearance of unconjugated serum BPA in adult humans, based on the assumption that the inability to detect unconjugated BPA with an insensitive assay indicated that all unconjugated BPA had been very rapidly metabolized. Thus, there has been strong criticism concerning the use of this one study as the basis for this prediction (Gies et al. 2009; Vandenberg et al. 2010a, 2010b). In the absence of data on the level of clearance of unconjugated BPA from human serum, it is an attractive option to use primates as surrogates to resolve questions about the relevance for humans of data from rodent studies.

Given the controversies and the unanswered questions about current levels of human external and internal exposure and the rate of BPA metabolism, the objective of the study reported here was to compare the level of clearance of unconjugated (biologically active) BPA in an experimental model with putative direct relevance to humans (rhesus monkeys), and in a model used in dozens of published reports of adverse effects due to exposure to low doses of BPA (the CD-1/ICR mouse). In experiment 1, we used isotope-dilution liquid chromatography–mass spectrometry (LC-MS) to determine the concentration of biologically active (unconjugated) as well as conjugated BPA in serum over the 24 hr after one or seven daily oral doses of 400 μg/kg/day deuterated BPA (dBPA) to adult female rhesus monkeys. Experiment 2, with adult female CD-1 mice, consisted of three parts. In experiment 2A, we administered a single 400 μg/kg/day oral dose of BPA, but we used ^3^H-BPA to ensure that we would be above the limit of quantitation (LOQ) throughout the 24 hr after administration. In experiment 2B, we used ^3^H-BPA to examine the linear relationship between administered oral dose and serum concentration of unconjugated ^3^H-BPA over a 50,000-fold dose range (2 μg/kg to 100,000 μg/kg). In experiment 2C, we examined the concentration of unconjugated and conjugated serum BPA over the 24 hr after administration of 100,000 μg/kg BPA and determined whether the results were 250-fold higher than those obtained using the 400 μg/kg/day dose of ^3^H-BPA. Finally, in experiment 3 we compared our data on conjugated serum BPA levels in monkeys and mice with prior published findings in adult women (Völkel et al. 2002). Our focus here is on unconjugated and conjugated BPA in serum. A more detailed analysis of BPA metabolites has been reported in CD-1 mice (Jaeg et al. 2004; Zalko et al. 2003) and is currently being conducted in rhesus monkeys.

## Materials and Methods

### Animals

All animals used in these studies were treated humanely and with regard for alleviation of suffering. All studies were conducted in accordance with National Institutes of Health guidelines (Institute of Laboratory Animal Resources 1996).

#### Monkeys

Eleven adult female rhesus macaques (*Macaca mulatta*) were housed at the California National Primate Research Center. Animals were caged individually with a 0600- to 1800-hour light cycle and at a temperature maintained at 25–27°C. Animals were fed a diet of Purina Monkey Chow (Purina-Mills, St. Louis, MO, USA) and water *ad libitum*. Seasonal produce, seeds, and cereal were offered as supplements for environmental enrichment. Cages were made of stainless steel, and water was delivered to each cage by rigid polyvinyl chloride pipes and a water nipple. Only females with a history of normal menstrual cycles were selected for this study. Females ranged in age from 6 to 13 years, and body weights ranged from 6.17 to 8.95 kg (mean, 7.5 kg). Cephalic vein blood samples were collected from unanesthetized, cage- restrained animals that were trained to present an arm for the procedure. Animal protocols were reviewed and approved in advance by the Animal Care and Use Committee of the University of California–Davis.

#### Mice

CD-1 mice were purchased from Charles River Laboratories (Wilmington, MA, USA) and maintained as an outbred stock (with periodic replacement) at the University of Missouri–Columbia. Animals were housed on corncob bedding in standard (11.5 × 7.5 × 5 in.) polypropylene cages. Water was purified by reverse osmosis and carbon filtration and provided in glass bottles *ad libitum*. Pregnant and lactating females were fed Purina soy-based 5008 breeder chow and otherwise were maintained on Purina soy-based 5001 maintenance chow (Purina-Mills). Rooms were kept at 25 ± 2°C under a 12:12-hr light:dark cycle. Animals were euthanized by CO_2_ asphyxiation and cervical dislocation, after which blood was collected from the carotid and vertebral vessels. Animal procedures were approved by the University of Missouri–Columbia Animal Care and Use Committee.

### Statistical methods for calculation of pharmacokinetic parameters

The following parameters were measured in monkeys and mice from the serum concentrations of BPA after oral administration. The *C*_max_ is the maximum concentration in serum. Our decision to use 0.5 hr as the first time of collection was based on the fact that in most prior studies this was reported as the time at which the maximum concentration was reached (*T*_max_). Our initial rate constant (*K*_initial_) was calculated from the slope of the natural log of the concentration versus the sample collection time. *K*_initial_ was taken as the steepest rate of decay from the initial collection time points. The terminal phase elimination rate constant (*K*_terminal_) was taken from the last three time points (between 4 and 24 hr for mice, between 8 and 24 hr for monkeys, and between 12 and 24 hr for humans). Half-lives (*t*_½_) were calculated as the natural log of 0.5 divided by the rate constant. The area under the curve (AUC) for the first 24 hr after dosing (AUC_0–24_) was calculated using the linear trapezoidal rule and the assumption that BPA in serum at the time just before administration (time 0) was zero. The AUC extrapolated to infinity (AUC_0→∞_) was calculated by dividing the concentration at 24 hr (the last time point measured) by the terminal rate constant and adding this term to the AUC_0–24_. We conducted day 1 and day 7 comparisons for serum BPA concentrations in experiment 1 using Proc Mixed analysis of variance (ANOVA) with repeated measures followed by least-squares means in SAS (version 6.12; SAS Institute Inc., Cary NC, USA).

### Experimental methods

#### Experiment 1: unconjugated and conjugated serum dBPA concentrations in rhesus monkeys

We used dBPA in experiment 1 because it can be clearly distinguished by isotope-dilution LC-MS, thus eliminating concern about potential BPA contamination from materials used in the preparation, handling, or shipment of samples. The monkeys were fed 400 μg/kg body weight of dBPA, chosen based on the oral dose estimated to be required to achieve an average dBPA 24-hr serum concentration in the range of 1–2 ng/mL, which is the range typically found in biomonitoring studies of adult men and women (Vandenberg et al. 2010a). The LOQ was 0.2 ng/mL based on analysis of dBPA in approximately 1.5 mL serum. See Supplemental Material, Part 1 (doi:10.1289/ehp.1002514) for details of LC-MS analysis.

Monkeys were fed 400 μg/kg body weight dBPA in food for 7 days. On the first and seventh days of feeding, blood was collected over 24 hr, with collection at 0 (prefeeding), 0.5, 1, 2, 4, 8, 12, and 24 hr after feeding dBPA (each collection yielded ~ 1.5 mL serum). Blood was allowed to stand at room temperature for about 15 min to clot (preliminary studies showed that no deconjugation of conjugated BPA occurred during this short time). Blood was then centrifuged at 1,800 × *g* for 10 min at 4°C. Serum was stored at −80°C and shipped overnight on dry ice from the University of California–Davis to the University of Missouri–Columbia. The assays were conducted at the University of Missouri Veterinary Diagnostic Laboratory.

#### Experiment 2A: unconjugated serum ^3^H-BPA concentrations in mice (400 μg/kg dose)

Serum concentrations of unconjugated ^3^H-BPA were examined in adult (~ 3 months of age) female CD-1 mice throughout the 24 hr after administration of a 400 μg/kg oral dose dissolved in tocopherol-stripped corn oil. The volume delivered into the animal’s mouth via a micropipetter (~ 30 μL) was adjusted to achieve a constant BPA dose per kilogram of body weight. Preliminary tests were performed to determine the volume of oil remaining in the pipette tip after dosing, and the total volume per mouse was adjusted to allow for this remaining amount. Mice were fed a 400 μg/kg dose of ^3^H-BPA instead of dBPA because of the limited amount of serum obtained from mice, which required a method with high sensitivity (Taylor et al. 2008). ^3^H-BPA (7.3 Ci/mmol, 3.0 μCi/dose; Moravek Biochemicals, Brea, CA, USA) was mixed with unlabeled BPA (> 99% pure; Sigma-Aldrich, St. Louis, MO, USA) to achieve a final estimated concentration of 12 μg BPA/30 μL. The actual concentration administered (12.1 μg/30 μL) and the specific activity (0.048 Ci/mmol) were determined from samples of the dosing solution. Blood was collected at 0.5, 1, 2, 3, 4, 6, and 24 hr after ^3^H-BPA administration, with five or six adult females at each time point. Serum was separated by centrifugation at 4°C and then stored at −20°C. Unconjugated ^3^H-BPA was measured in serum as described in the Supplemental Material, Part 1 (doi:10.1289/ehp.1002514).

#### Experiment 2B: relationship between BPA dose and unconjugated serum BPA concentration in mice

Adult (~ 3 months of age) female CD-1 mice were administered a single oral dose of ^3^H-BPA mixed with different amounts of unlabeled BPA in tocopherol-stripped corn oil to achieve administered oral doses of 2, 20, 400, or 100,000 μg/kg body weight in approximately 30 μL oil. Specifically, ^3^H-BPA was mixed with unlabeled BPA (> 99% pure; Sigma-Aldrich) to achieve the final concentrations. Samples of each solution were kept to measure the actual radioactivity used in each dose; the final specific activities for each dose were calculated from these aliquots rather than from the theoretical radioactivity per dose. The measured specific activities of the 2, 20, 400, and 100,000 μg/kg solutions were 7.30, 0.87, 0.04, and 0.0002 Ci/mmol, respectively, and the actual doses administered were 2.3, 20.1, 396.9, and 98,447 μg/kg, respectively. Because BPA was not soluble in oil at the highest concentration (120 mg/mL), it was instead administered as a suspension; radioactivity in this suspension was comparable to that in the highest soluble concentration, as anticipated. Blood was collected 24 hr after treatment, and serum was separated by centrifugation at 4°C and then stored at −20°C until analysis for unconjugated ^3^H-BPA.

#### Experiment 2C: unconjugated and conjugated serum BPA concentrations in mice (100,000 μg/kg dose)

Adult (~ 3 months of age) female CD-1 mice (four per group) were given a single oral dose of BPA (> 99% pure; Sigma-Aldrich) via a micropipetter. The volume administered (~ 30 μL) was adjusted to achieve a constant 100,000 μg BPA dose per kilogram of body weight. Blood was collected at 0, 0.5, 1, 2, 3, 4, 6, or 24 hr after administration, and serum was separated by centrifugation at 4°C. Serum from the four mice in each group at each time point was pooled, and samples were stored at −20°C until analysis for unconjugated and conjugated BPA by high-performance liquid chromatography (HPLC) with CoulArray detection (CoulArray 5600 detector; ESA, Chelmsford, MA, USA). See Supplemental Material, Part 1 (doi:10.1289/ehp.1002514) for assay details.

#### Experiment 3: comparison of results from adult female monkeys and mice with data from women

We compared results from experiments 1 and 2C with data from a study by Völkel et al. (2002), which involved a single oral administration of dBPA (average administered dose, 69.3 μg/kg) to adult men and women. The authors reported data for conjugated serum dBPA during the 24 hr after the single oral administration. We scaled the dose administered to monkeys to the dose administered to humans [based on accepted linearity of BPA pharmacokinetics with dose (Doerge et al. 2010a; Vandenberg et al. 2007)] by multiplying the monkey serum-conjugated dBPA values at each time point by a factor of 0.173 (69.3/400 μg/kg). We scaled the dose administered to mice (a single 100,000 μg/kg dose of BPA) to the dose administered to humans by multiplying the mouse serum–conjugated BPA values at each time point by a factor of 0.000693 (69.3/100,000 μg/kg). We used GraphClick (version 3.0; Arizona Software 2008) to capture data and calculate the AUC for women by integration of the curve fit equation presented by Völkel et al. (2002) in their Figure 7.

## Results

### Experiment 1: unconjugated and conjugated serum dBPA concentrations in rhesus monkeys

In this experiment, we determined the concentrations of unconjugated and conjugated dBPA in 11 adult female rhesus monkeys over a 24-hr period after a single oral 400 μg/kg dose of dBPA and compared the data after one administration with data from the same animals after seven daily oral administrations. The results for unconjugated and conjugated dBPA on days 1 and 7 (Figure 2) reveal that the serum levels of unconjugated dBPA were very similar after a single oral dose and after seven doses, indicating that bioaccumulation of parent dBPA did not occur in response to a single oral exposure each day (the AUC_0–24_ was virtually identical for days 1 and 7; Table 1). Our findings show that the maximum attained value (*C*_max_) for unconjugated dBPA in serum at 1 hr after feeding was 3.95 ng/mL on day 1 and was 4.40 ng/mL on day 7 (Table 1). By 24 hr after administration, unconjugated dBPA remained above our LOQ (0.2 ng/mL; ppb) for 5 of the 11 females on day 1 and for 4 of 11 females on day 7. The AUC_0–24_ for unconjugated serum dBPA on day 1 was 12.36 ng-hr/mL and on day 7 was 11.47 ng-hr/mL. The *K*_terminal_ for conjugated dBPA on day 7 (−0.04/hr) was somewhat slower than on day 1 (−0.07/hr). Only at 1 hr after oral administration was the concentration of conjugated serum dBPA significantly higher on day 7 than on day 1 (*p* < 0.005; Figure 2). Over all time points, however, we observed no significant difference between day 1 and day 7 in serum-conjugated dBPA. The AUC_0–24_ ratio for conjugated/unconjugated serum dBPA was 116 on day 7 and 87 on day 1.

### Experiment 2A: unconjugated serum ^3^H-BPA concentrations in mice (400 μg/kg dose)

In this experiment we determined the serum concentration of unconjugated ^3^H-BPA in adult female CD-1 mice over the 24 hr after oral administration of the same 400 μg/kg dose used in experiment 1 with adult female rhesus monkeys. The serum concentration of unconjugated ^3^H-BPA in the mice is shown in Figure 3 in relation to the data from experiment 1 for unconjugated dBPA in female rhesus monkeys over the 24-hr time period after treatment. The calculated parameters for the mice are shown in Table 2. For unconjugated serum ^3^H-BPA in mice, the *C*_max_ was 3.28 ng/mL at 1 hr (*T*_max_). AUC_0–24_ for unconjugated ^3^H-BPA was 16.72 ng-hr/mL, a low value that was similar to the value obtained for the monkeys administered the same dose and time period (12.36 ng-hr/mL). Because we did not have an authentic standard for either BPA glucuronide or BPA sulfate, the two expected BPA conjugates, we did not attempt to quantify conjugated serum ^3^H-BPA in this experiment.

### Experiment 2B: relationship between ^3^H-BPA dose and unconjugated serum ^3^H-BPA concentration in mice

The objective of experiment 2B was to determine the relationship between administered oral dose and serum concentration of ^3^H-BPA in adult female CD-1 mice measured 24 hr after BPA dosing. In more detail, the results shown in Figure 4 reveal that oral administration of a single dose of ^3^H-BPA at 2–100,000 μg/kg resulted in a linear relationship (*R*^2^ = 0.9807) between the administered dose and the serum concentration of unconjugated ^3^H-BPA 24 hr after administration (based on a log–log plot). Thus, these results provide evidence for a linear relationship between doses and unconjugated serum BPA concentrations in mice.

### Experiment 2C: unconjugated and conjugated serum BPA concentrations in mice fed a single dose of 100,000 μg/kg

In experiment 2B we observed a linear relationship between the administered dose of BPA and unconjugated serum BPA over a 50,000-fold dose range (2–100,000 μg/kg). Here we sought to determine whether adult female CD-1 mice fed a 100,000 μg/kg dose showed the serum concentrations of unconjugated BPA predicted by linear extrapolation when adjusted to a dose of 400 μg/kg by dividing all serum concentrations by a scaling factor of 250. Because of the high dose administered, instead of ^3^H-BPA we used a chemical analysis method (HPLC with CoulArray detection) to determine the unconjugated and conjugated concentrations of BPA. This approach allowed comparison of the use of ^3^H-BPA and authentic BPA on determination of serum concentrations of BPA over the 24 hr after oral administration.

The average values for unconjugated and conjugated BPA over the 24 hr after a single oral dose of 100,000 μg/kg are shown in Figure 5 and Table 2. When we extrapolated (scaled) the 100,000 μg/kg dose to 400 μg/kg (by dividing each serum BPA value by 250) for comparison with the data from adult female mice administered 400 μg/kg ^3^H-BPA, unconjugated serum values of BPA and ^3^H-BPA over the 24 hr after a single feeding were not significantly different (Figure 6). This finding reveals that the data for ^3^H-BPA determined by HPLC separation and scintillation counting were virtually identical to what would be predicted based on analysis of BPA by HPLC with CoulArray detection. This finding also provides additional evidence for linearity between administered dose and unconjugated serum BPA in adult female mice throughout the entire 24-hr period after oral administration.

### Experiment 3: serum concentrations of conjugated BPA in monkeys and mice compared with data from women

The study by Völkel et al. (2002) involved a single oral dose of dBPA (average, 69.3 μg/kg) to adult men and women. The assay the authors used lacked the sensitivity required to measure unconjugated dBPA; thus, they reported only data for conjugated serum dBPA during the 24 hr after the single oral administration (Völkel et al. 2002). Because we observed a linear relationship between administered BPA dose and serum levels of BPA in adult female mice in experiments 2B and 2C using two different approaches, and because dose proportionality for total serum BPA has also been reported in rats (Doerge et al. 2010b), we compared the data by Völkel et al. (2002) for serum-conjugated dBPA in women with our data for rhesus monkeys and CD-1 mice. The BPA dose administered to monkeys was scaled to the human dose by multiplying the monkey serum-conjugated dBPA values at each time point by 0.173 (69.3/400 μg/kg). The BPA dose administered to mice, from experiment 2C in which mice were fed a single 100,000 μg/kg dose of BPA, was scaled to the human dose multiplying the mouse serum-conjugated BPA values at each time point by a factor of 0.000693 (69.3/100,000 μg/kg).

We used only the data for women reported by Völkel et al. (2002) (these are the only available data for women) because rodent data suggest that sex differences related to background levels of testosterone may alter the metabolism of BPA (Shibata et al. 2002; Takeuchi et al. 2006) and because there are differences in total BPA in urine btween men and women (Calafat et al. 2008). In addition, the data of Völkel et al. (2002) differed for men and women at 24 hr (see their Figure 7).

The data comparing women, adult female monkeys, and adult female mice, presented in Figure 7 and Table 3, reveal that for the women examined by Völkel et al. (2002) and the adult female rhesus monkeys and mice that we examined, the kinetics were very similar for conjugated BPA in serum. In calculating the AUC data in Table 3, we used only data between 4 and 24 hr for women, monkeys, and mice because Völkel et al. (2002) did not report results for women before 4 hr. Therefore, we were also able to compare directly only the *K*_terminal_ values and not the *K*_initial_ values. However, in Figure 7 we show all of our data for rhesus monkeys and mice, including the results for time points before 4 hr, although data from collections before 4 hr were not used in the analyses shown in Table 3.

The SE for serum-conjugated dBPA for women and female monkeys overlapped at every time point examined (Figure 7). The absence of a difference in these data among women, monkeys, and mice was reflected in the similarity in values for the AUC between 4 and 24 hr after feeding (AUC_4–24_; Table 3). Importantly, the data for mice were similar to those for women and monkeys between 4 and 24 hr after a single feeding (Figure 7, Table 3).

## Discussion

In this study in rhesus monkeys, an experimental model with direct relevance to humans, we assessed the serum concentrations of unconjugated (biologically active) and conjugated dBPA over the 24-hr period after oral exposure to 400 μg/kg dBPA predicted on the basis of biomonitoring studies to be relevant to human exposure levels (Vandenberg et al. 2007, 2010b; vom Saal et al. 2007). We then evaluated the relevance of a rodent model for primates by comparing the level of clearance of unconjugated BPA from serum in the mouse compared with the rhesus monkey. Because marked differences between rodents and primates have been predicted (Goodman et al. 2009), these experiments directly address two central issues that have been controversial: *a*) the rate at which unconjugated BPA is cleared from serum in rhesus monkeys and mice, and *b*) the oral dose of BPA necessary in rhesus monkeys and mice to achieve serum levels of unconjugated BPA found in numerous biomonitoring studies in humans.

### Metabolism of oral BPA in monkeys and mice

**Figure 1:**
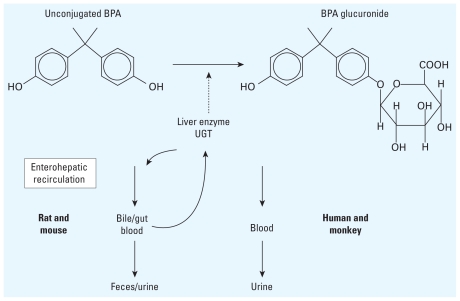
Schematic diagram depicting the glucuronidation of BPA in the liver and the route of elimination of unconjugated BPA from serum in rodents and primates after initial absorption from the gut and transport to the liver. There is evidence that enterohepatic recirculation in rodents has only a modest impact on unconjugated serum BPA (Pottenger et al. 2000).

An often-stated assumption is that humans rapidly conjugate all ingested BPA, primarily via the action of UGT (Figure 1) during the first pass of BPA through the liver. (BPA is rapidly absorbed from the intestines and transported to the liver via the portal vessels leading directly from the gut to the liver.) Of great importance, our findings demonstrate that the first-pass metabolism of parent BPA after oral administration in rhesus monkeys is not rapid or complete. In addition, our results show that the mean unconjugated serum dBPA concentrations at both 8 hr (0.35 ng/mL) and 12 hr (0.15 ng/mL) after one oral administration of 400 μg/kg dBPA were both well within the biologically active range of BPA in human tissues and cells (Hugo et al. 2008; Wetherill et al. 2002).

These data directly contradict statements made in reviews funded by the Polycarbonate/BPA Global Group (Dekant and Völkel 2008; Goodman et al. 2009). For example, Goodman et al. (2009) stated that “orally administered BPA is subject to extensive (≥ 99%) first-pass detoxifying metabolism.” These authors cited Völkel et al. (2002) as the basis for the conclusion that there was little concern for human health due to exposure to BPA. The prediction of rapid and complete first-pass elimination of parent BPA in adult humans is based on a single study of BPA metabolism in humans after one oral dose (Völkel et al. 2002). In that study using three women and six men, the LOQ was > 10 times higher than in other published studies using the same techniques (reviewed by Vandenberg et al. 2010a). Because the assay used by Völkel et al. (2002) was unable to detect unconjugated BPA in serum, the authors made predictions regarding the kinetics of unconjugated BPA in the absence of data. We also note that if the data presented in Figure 7 of Volkel et al. (2002) are reanalyzed with the inclusion of the 24-hr time point for men (a value that was excluded without explanation) and using conjugated rather than total BPA values for all time points, the terminal half-life increases from the reported value of 3.4 hr to 6.0 hr. Our results thus provide compelling evidence that assumptions about the rate of BPA metabolism in humans based on the study by Völkel et al. (2002) are inaccurate; this is consistent with similar conclusions reached by others (Gies et al. 2009; Vandenberg et al. 2010b).

### Oral doses of BPA required to achieve measured human serum levels

The second major issue of contention concerns estimates regarding the amount, as well as the route of exposure, required to account for BPA levels between 0.3 and 4 ng/mL detected in human serum and urine in biomonitoring studies. The prediction that intermittent oral exposure accounts for virtually all exposure to BPA by adults is clearly not consistent with these findings or a large number of other published studies (Vandenberg et al. 2010a). Specifically, in our study with rhesus monkeys, we were required to administer a relatively high (400 μg/kg) dBPA oral dose compared with predicted human BPA oral exposure of < 1 μg/kg/day to achieve serum concentrations similar to those reported in biomonitoring studies. However, our dBPA dose resulted in a relatively low 24-hr average serum concentration of bioactive (unconjugated) dBPA (0.52 ng/mL) and a maximum value of 3.95 ng/mL 1 hr after administration. These findings should be considered in relation to numerous biomonitoring studies reporting median levels of 0.3–4 ng/mL unconjugated BPA in serum from men and women (Vandenberg et al. 2010a).

Only a few authors have rejected data from human biomonitoring studies (Dekant and Völkel 2008; Doerge et al. 2010b; Goodman et al. 2009). This rejection is based on the assumption that data demonstrating BPA levels inconsistent with exposure models that presume that humans ingest < 1 μg/kg/day BPA must have involved the use of contaminated equipment that was the source of the measured BPA. Although those making this claim report substantial laboratory BPA contamination in the range of ≥ 2 ng/mL (Doerge et al. 2010a; Völkel et al. 2002), most of the studies being rejected included explicit and appropriate controls for contamination and measured and reported low to undetectable background BPA, and thus a low LOQ, which is also the case for our studies (reviewed by Vandenberg et al. 2010b). Several of the studies also detailed the steps taken to achieve a low to undetectable background contamination. Thus, other reasons, such as selectivity of the analytical technique, would be required to support the hypothesis of overestimation of human plasma BPA levels.

Our findings thus provide experimental support for the prediction made in the National Institutes of Health–sponsored Chapel Hill Consensus Statement (Vandenberg et al. 2007; vom Saal et al. 2007) that, to account for the published concentrations of unconjugated serum BPA in adult men and women, daily oral doses of BPA would have to be at least 500 μg/kg (Vandenberg et al. 2007, 2010b). The high end of the range of median values reported for unconjugated BPA in human serum corresponds to the highest levels we saw only briefly in rhesus females after the oral administration of 400 μg/kg/day, a dBPA dose 8 times higher than the current U.S. Environmental Protection Agency’s “safe” daily intake dose of 50 μg/kg/day (U.S. EPA 1988). Thus, if serum BPA concentrations in humans are actually between 0.3 and 4 ng/mL, our data raise grave concern that regulatory agencies have grossly underestimated current human exposure levels because they have relied on the prediction of Völkel et al. (2002) that nearly total first-pass metabolism will ensure that bioactive BPA is not present in human sera, when in fact multiple human biomonitoring studies have established this to be false.

On the basis of our findings, we propose that the higher-than-predicted serum levels of unconjugated BPA in men and women reflect significant nonoral BPA exposure in addition to oral exposure. This is consistent with other evidence suggesting that the consumption of BPA-contaminated food and beverages alone is insufficient to account for the BPA levels reported in human biomonitoring studies (Vandenberg et al. 2010b); this includes data from the National Health and Nutrition Examination Survey (NHANES) conducted by the Centers for Disease Control and Prevention (Stahlhut et al. 2009). A significant data gap is the absence of a comprehensive list of products containing BPA. Of particular concern is information about sources of nonoral exposures that would lead to higher serum BPA concentrations relative to oral exposures (no hepatic first-pass effect), because unconjugated serum BPA levels are higher in adults after nonoral exposure than after oral exposure (Vandenberg et al. 2007). One example of a recently identified source of human exposure to BPA is thermal paper receipts that could potentially result in transdermal exposure (Biedermann et al. 2010).

### Kinetics of metabolism in monkeys and mice, and comparison with prior data from women

After an oral BPA dose of 400 μg/kg, the serum concentrations of BPA in adult female CD-1 mice and rhesus monkeys were very similar. However, the average concentration of unconjugated BPA in serum over the 24 hr after administration to both mice and rhesus monkeys (based on the average AUC_0–24_) was about 0.5 ng/mL, which is at the low end of the median concentrations of unconjugated serum BPA (range, 0.3–4.4 ng/mL, or 1–19.4 nM) in men and women (Vandenberg et al. 2010a). These findings thus contradict an important assumption made by U.S. and European regulatory agencies, namely, that rodents and primates are predicted to show markedly different clearance levels of BPA from serum. Importantly, this assumption has been central to the argument that rodent studies are not relevant to primates (including humans) for assessing the safety of BPA (reviewed by Gies et al. 2009). Our data (Figure 7) demonstrate the similarity in the rate of phase II BPA metabolism (based on conjugated BPA in serum) for humans, rhesus monkeys, and mice.

**Figure 2:**
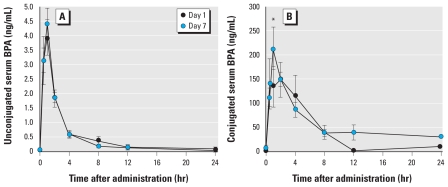
Concentrations (mean ± SE) of unconjugated (*A*) and conjugated (*B*) dBPA in serum from adult female rhesus monkeys during the 24 hr after oral administration of 400 μg/kg body weight. Data represent the time course on day 1 (after one dose) and day 7 (after seven daily doses); *n* = 8–11 monkeys per time point. *p < 0.005 for day 1 compared with day 7.

**Figure 3:**
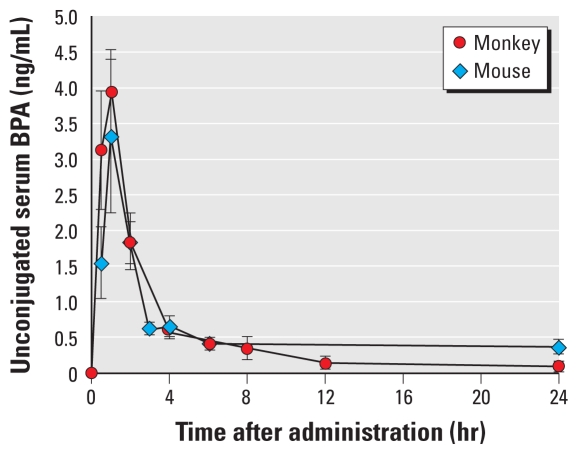
Concentrations (mean ± SE) of unconjugated serum BPA during the 24 hr after oral administration of 400 μg/kg ^3^H-BPA to adult female CD-1 mice (*n* = 5–7 per time point) and 400 μg/kg dBPA to adult female rhesus monkeys (*n* = 10–11 per time point).

**Figure 4:**
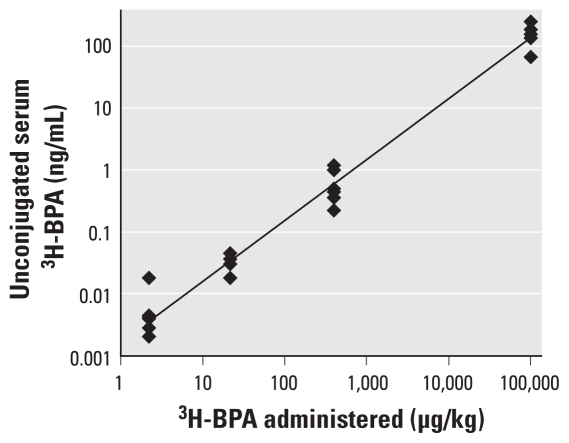
Concentration of unconjugated serum ^3^H-BPA in adult female CD-1 mice in relation to the administered oral dose of BPA over a 50,000-fold dose range (nominal dose: 2, 20, 400, and 100,000 μg/kg). Blood was collected 24 hr after administration of BPA. *y* = 0.0017*x*^0.9798^; *R*^2^ = 0.9807.

**Figure 5:**
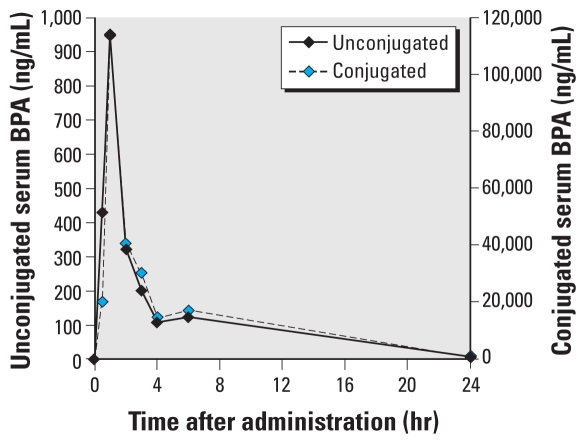
Unconjugated and conjugated serum BPA concentrations in adult female CD-1 mice (*n* = 4 per time point) during the 24 hr after a single oral dose of BPA (100,000 μg/kg).

**Figure 6:**
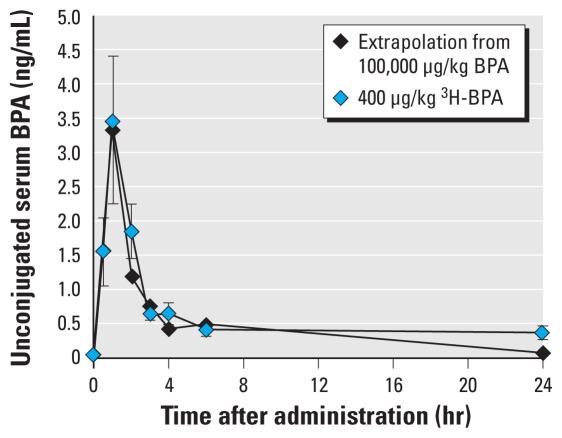
Concentration of unconjugated serum BPA in adult female CD-1 mice during the 24 hr after a single dose of 400 μg/kg ^3^H-BPA or 100,000 μg/kg BPA. The data for the 100,000-μg/kg dose are extrapolated (scaled) to the 400 μg/kg data based on the demonstrated linear relationship between serum BPA and dose administered (Figure 4).

Some authors have emphasized the importance of enterohepatic recirculation in rodents as a critical factor that results in higher serum levels of unconjugated BPA relative to primates after a similar oral dose (Teeguarden et al. 2005). In fact, the data presented here (Figures 3 and 5) show a very slight increase in unconjugated serum BPA in adult female mice (but not rhesus monkeys; Figure 2) between 4 and 6 hr after oral administration of BPA at 400 μg/kg and 100,000 μg/kg. A similar small but not statistically significant increase in unconjugated serum BPA between 4 and 6 hr after oral administration of BPA in rats has been reported by others (Pottenger et al. 2000).

Taken together, the data do not support the contention that enterohepatic recirculation of BPA is a major factor that justifies disregarding findings from rodent studies in assessing the potential risks to humans posed by doses of BPA thousands of times lower than the assumed lowest observed adverse effect level of 50 mg/kg/day, the level that was used to calculate the reference dose of 50 μg/kg/day (U.S. EPA 1988). In addition, the recognized difference in route of clearance of BPA between rodents (primarily via the feces) and primates (primarily via the urine) has also been incorrectly interpreted as supporting the prediction of a different level of clearance of BPA. Our data clearly demonstrate that, in rhesus monkeys and mice, the rate of clearance of unconjugated BPA from serum during the 24 hr after oral BPA administration is virtually identical. These findings are consistent with those of Tominaga et al. (2006), who reported pharmacokinetic differences between cynomolgus monkeys and rats during the first 4 hr after BPA administration but no difference in the 24-hr average BPA serum concentration (based on the average AUC_0–24_).

### Relationship between administered and internal dose of BPA, and age-related changes in BPA pharmacokinetics

Our studies also provide data on the relationship between administered dose of BPA and unconjugated serum BPA. In adult female mice, we found this relationship to be linear over a wide range of administered oral doses (particularly between 2 and 400 μg/kg). This finding was predicted based on numerous studies in rats (Vandenberg et al. 2007). A practical reason for examining this relationship is that researchers need to know whether it is necessary to determine the internal concentration of BPA for every dose administered or if they are potentially able to extrapolate from data obtained with a high dose to predict internal dose in response to low administered doses of BPA; it is difficult to measure very low concentrations of BPA in the limited amount of serum obtained from mice or any young rodent. Our previous study (Taylor et al. 2008), in which we compared unconjugated serum ^3^H-BPA after oral administration and subcutaneous injection in newborn mice (where it is difficult to measure very low concentrations of BPA in the limited amount of serum obtained), also revealed linearity of serum BPA with administered dose regardless of route of administration.

A final important issue concerns the comparison of BPA metabolism in infant and adult rodents and rhesus monkeys. We previously reported that the AUC_0–24_ for 3-day-old CD-1 female mice fed 395 μg/kg/day ^3^H-BPA in oil was 66.7 ng-hr/mL, with a *C*_max_ of 14.8 ng/mL (Taylor et al. 2008). Our data here show a 4-fold decrease in the AUC and a 4.5-fold decrease in *C*_max_ in adult female CD-1 mice administered an oral dose of 400 μg/kg BPA (Table 2), reflecting the more rapid metabolism of BPA in adults relative to newborn mice. Consistent with these findings, UGT activity toward BPA between postnatal days 3 and 21, when adult levels of metabolism are reached, was shown to increase 4-fold in Wistar rats, which would result in adults conjugating BPA 4 times faster than infants (Matsumoto et al. 2002).

In contrast to our findings with CD-1 mice, Doerge et al. (2010a) reported a markedly different change in the rate of unconjugated BPA clearance between birth and adulthood in the FDA National Center for Toxicological Research’s (NCTR) CD-SD rat, with a 20.5-fold decrease in AUC and a 74.4-fold decrease in *C*_max_ for unconjugated BPA between postnatal day 3 and adulthood. In a companion study with rhesus monkeys, Doerge et al. (2010b) also provided evidence for an age-related decrease in AUC (3.8-fold) and *C*_max_ (2.7-fold) for unconjugated BPA between 5-day-old rhesus monkeys and adults, changes similar in magnitude to those in CD-1 mice based on data in the present study and our previous study (Taylor et al. 2008). However, the rhesus monkey study by Doerge et al. (2010b) involved a small number of animals, and the age-related differences were reported to not reach statistical significance. Thus, although Doerge et al. (2010b) found evidence for approximately a 4-fold change in the rate of metabolism of unconjugated BPA between infants and adults after oral exposure in rhesus monkeys, they concluded that “there was no evidence for diminished Phase II metabolism” in infants.

**Figure 7:**
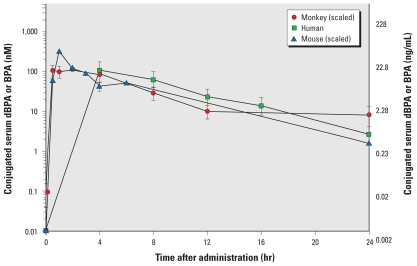
Concentration of conjugated dBPA or BPA in serum from adult female rhesus monkeys, CD-1 mice, and humans during the 24 hr after one oral dose. Women were administered an average dose of 69.3 μg/kg dBPA (Völkel et al. 2002). Rhesus monkeys were administered 400 μg/kg dBPA and mice were administered 100,000 μg/kg BPA; results for monkeys and mice were scaled to 69.3 μg/kg, based on evidence for linear kinetics and because in mice the administered dose was linear with serum BPA between 2 and 100,000 μg/kg (Figure 4). Both nanomolar and nanograms per milliliter data are presented for comparison with the human data of Völkel et al. (2002).

In the present study and in our prior study in neonatal mice (Taylor et al. 2008), we used CD-1/ICR mice, the model animal used by the National Toxicology Program and in > 20 published studies from different laboratories reporting adverse effects of BPA (reviewed by Myers et al. 2009; Richter et al. 2007). The conclusion by Doerge et al. (2010b) that “pharmacological effects observed in early postnatal rats could overpredict those possible in primates of the same age” may thus be accurate only for the NCTR CD-SD strain of rat, a strain derived from the CD-SD rat (Latendresse et al. 2009) that, in contrast to the CD-1 mouse, has not shown low-dose effects of BPA in many toxicological studies (reviewed by vom Saal and Hughes 2005). Our present findings clearly demonstrate that adult CD-1 mice and rhesus monkeys show virtually identical clearance of unconjugated BPA from serum over the 24 hr after a single oral administration, and that both the mouse and the monkey are very similar to humans in serum-conjugated BPA over the 24 hr after administration of the same dose (Figure 7). Our findings support the consensus report on BPA from a meeting held by the German Federal Environment Agency (Umweltbundesamt) (Gies et al. 2009) that rodents are appropriate models for predicting serum levels of bioactive BPA in primates.

Many claims have been made concerning the lack of relevance of rodents for predicting the consequences of BPA exposure for primates, including humans. A large number of low-dose studies reporting adverse effects of BPA in mice have involved administered doses that our findings here and elsewhere (Taylor et al. 2008) show result in internal doses of unconjugated BPA that are already far exceeded by those found in multiple biomonitoring studies in humans (reviewed by Richter et al. 2007; Vandenberg et al. 2007, 2010a). For example, based on linearity of administered and internal dose, a 20 μg/kg oral dose of BPA is predicted to lead to an average serum concentration over 24 hr of about 0.04 ng/mL BPA in adult CD-1 mice (Table 2). This 20 μg/kg/day oral dose of BPA caused adverse effects in adult mice as well as in adult rats (Alonso-Magdalena et al. 2006; Bindhumol et al. 2003; Sakaue et al. 2001; reviewed by Richter et al. 2007). Assertions that low-dose rodent studies involving both developmental and adult exposures are irrelevant for predicting the risk posed by BPA to human health are misguided. These assertions also ignore a large body of literature showing that BPA has equal potency in both rodent and human cells (Welshons et al. 2006).

## Conclusions

Many studies have attempted to portray the inability to detect unconjugated serum BPA in one experiment conducted with a limited sample size and a relatively insensitive assay (Völkel et al. 2002) as an indication that all administered BPA is completely metabolized during its first pass through the liver. Our findings with rhesus monkeys in the present study do not support this conclusion and indicate that the adult rhesus monkey is a valid model for predicting the serum levels of conjugated BPA after oral exposure in humans. Our findings also suggest that the mouse is a valid predictor of serum-conjugated BPA after oral exposure in humans. Finally, when the data on BPA metabolism in infant and adult rhesus monkeys reported in an FDA study (Doerge et al. 2010b) are compared with our findings in neonatal CD-1 mice (Taylor et al. 2008) and our data presented here, virtually identical age-related changes in the rate of metabolism of unconjugated BPA are evident in rhesus monkeys and CD-1 mice. These findings lead to the conclusion that the CD-1 mouse is a valid predictor of age-related changes in the rate of metabolism of BPA in rhesus monkeys and thus also likely in humans. Finally, ingestion of the currently estimated exposure level of BPA from food and beverages in the United States (0.16 μg/kg/day) is not consistent with our finding here of an average serum-unconjugated BPA concentration of about 0.5 ng/mL in rhesus monkeys and mice during the 24 hr after ingestion of 400 μg/kg/day BPA.

## Correction

In the manuscript originally published online, Pierre-Louis Toutain and Céline M. Laffont were omitted from the list of authors; there were calculation errors in Tables 1–3; and the y-axis for unconjugated BPA in Figure 5 was incorrect. All of these have been corrected here.

In Supplemental Material, Part 2 (doi:10. 1289/ehp.1002514), the authors have included the original data as mean, SE, and number of animals per treatment group, as well as analy sis of the data from these experiments using WinNonlin (Pharsight Corporation, Cary, NC, USA) and NONMEM (ICON Development Solutions, Ellicott City, MD, USA) soft-ware that is used by the Food and Drug Administration for analyzing pharmacokinetic data.

## Figures and Tables

**Table 1:** Kinetic parameters for unconjugated and conjugated serum dBPA in adult female rhesus monkeys during the 24 hr after one (day 1) or seven (day 7) oral doses of dBPA (400 μg/kg).

	Unconjugated		Conjugated	
Parameter	Day 1	Day 7	Day 1	Day 7
*C*_max_ (ng/mL)	3.95	4.40	149.47	226.96
*T*_max_ (hr)	1	1	2	1
*K*_initial_ (/hr)	−0.70	−0.86	−0.26	−0.41
Initial *t*_½_ (hr)	0.99	0.81	2.64	1.69
*K*_terminal_ (/hr)	−0.08	−0.10	−0.07	−0.04
Terminal *t*_½_ (hr)	8.88	7.20	10.08	17.92
AUC_0–24_ (ng-hr/mL)^a^	12.36	11.47	1068.67	1326.76
AUC_0_→∞ (ng-hr/mL)	13.44	11.87	1222.02	1893.75
Average AUC_0–24_ (ng/mL)	0.52	0.48	44.53	55.28

a Conjugated:unconjugated AUC_0–24_ ratios: day 1, 86.47; day 7, 115.70.

**Table 2:** Kinetic parameters for serum BPA in adult female CD-1 mice during the 24 hr after a single oral dose of 400 μg/kg or 100,000 μg/kg ^3^H-BPA.

Parameter	400 μg/kg dose (unconjugated)	100,000 μg/kg dose	
		Unconjugated	Conjugated
*C*_max_ (ng/mL)	3.28	949.14	114151.86
*t*_max_ (hr)	1	1	1
*K*_initial_ (/hr)	−0.71	−0.77	−0.66
Initial *t*_½_ (hr)	0.97	0.90	1.05
*K*_terminal_ (/hr)	−0.02	−0.14	−0.17
Terminal *t*_½_ (hr)	33.64	4.90	4.07
AUC_0–24_ (ng-hr/mL)^a^	16.72	2936.37	367887.45
AUC_0–∞_ (ng-hr/mL)	38.72	2990.87	371418.70
Average AUC_0–24_ (ng/mL)	0.70	122.35	15328.64
Scaled average AUC_0–24_ (ng/mL)^b^		0.49	61.32

a Conjugated/unconjugated AUC_0–24_ ratio = 125.29 ng-hr/mL.

b AUC 100,000 μg/kg was scaled to 400 μg/kg by dividing by 250.

**Table 3:** Kinetic parameters for conjugated dBPA in serum during the 24 hr after administration of 69.3 μg/kg dBPA to adult women (Völkel et al. 2002), compared with data from rhesus monkeys and CD-1 mice in the present study.

Kinetic parameter, day 1	Women	Monkeys	Mice
Concentration at 4 hr [ng/mL (SE)]	24.05 (9.52)	19.82 (7.52)	10.17
*K*_terminal_ (/hr)	−0.18	−0.07	−0.17
Terminal *t*_½_ (hr)	3.76	10.08	4.07
AUC_4–24_ [ng-hr-mL (SE)]	148.51 (25.42)	96.91 (18.91)	134.1
Average AUC_4–24_ (ng/mL)	7.43	4.85	6.7

The terminal *t*_½_ in women (*n* = 3) is based on data from Völkel et al. (2002; see their Figure 7) and is expressed in hours instead of minutes. The *K*_terminal_ was from 16 to 24 hr for women, 12 to 24 hr for monkeys, and from 6 to 24 hr for mice. Data presented here are for between 4 and 24 hr because Völkel et al. (2002) did not report data for women before 4 hr. Monkey and mouse data were scaled to 69.3 μg/kg from the single dose of 400 μg/kg dBPA fed to monkeys and 100,000 μg/kg BPA fed to mice. No variance estimates (SEs) are available from the mouse study (experiment 2C) because serum samples were pooled for each time point.
